# CONTACT FREQUENCY WITH CHILDREN FOLLOWING RELOCATION LATER IN LIFE: DO CONTACT MODES AND PROXIMITY TO A CHILD MATTER?

**DOI:** 10.1093/geroni/igad104.0173

**Published:** 2023-12-21

**Authors:** Joonyoung Cho, Ruth Dunkle, Jacqui Smith

**Affiliations:** University of Michigan, Ann Arbor, Michigan, United States; University of Michigan, Ann Arbor, Michigan, United States; University of Michigan, Ann Arbor, Michigan, United States

## Abstract

Contact with children is an important source of support for older adults. It is not clear if relocation and proximity to a child affect in-person communication as well as telephone, email, and social media contact between older parents and children. Using data from the Health and Retirement Study (2014-2018: N=3,332) we examined changes in communication frequencies between parents who either stayed or relocated in the last 4 years and potentially changed their close proximity to a child. We found differential effects of relocation on contact frequency depending on contact modes and proximity. Regarding in-person contact, unadjusted and adjusted linear regressions revealed older adults were less likely to meet up with their children regardless of the distance moved when a child was not nearby. In contrast to stayers, movers with proximity to a child were more likely to meet up with their children (adj. β=0.50, p ≤0.001). We found the same trend for telephone and email contact frequency. Movers with proximity to a child were more likely to have telephone (adj. β=0.17,p=0.034) and email contact (adj. β=0.33, p=0.023). Unlike other types of contact modes, social media contact frequency was not influenced by relocation or child proximity. While older adults tend to constrict their relationships, this trend is not applicable to every older adult. In the social convey model, situational characteristics (e.g., relocation) is considered, and heterogeneity in social relations in later life can be explained.

